# Molecular Transport within Polymer Brushes: A FRET View at Aqueous Interfaces

**DOI:** 10.3390/molecules27093043

**Published:** 2022-05-09

**Authors:** Quinn A. Besford, Simon Schubotz, Soosang Chae, Ayşe B. Özdabak Sert, Alessia C. G. Weiss, Günter K. Auernhammer, Petra Uhlmann, José Paulo S. Farinha, Andreas Fery

**Affiliations:** 1 Leibniz-Institut für Polymerforschung e.V., Hohe Str. 6, 01069 Dresden, Germany; Schubotz@ipfdd.de (S.S.); Chae@ipfdd.de (S.C.); besford-alessia@ipfdd.de (A.C.G.W.); auernhammer@ipfdd.de (G.K.A.); uhlmannp@ipfdd.de (P.U.); 2 Molecular Biology and Genetics Department, Istanbul Technical University, 34469 Istanbul, Turkey; ozdabak@itu.edu.tr; 3 Centro de Química Estrutural, Department of Chemical Engineering, Instituto Superior Técnico, Universidade de Lisboa, 1049-001 Lisboa, Portugal; farinha@tecnico.ulisboa.pt

**Keywords:** polymer brushes, FRET, molecular transport, spatially-resolved, wettability

## Abstract

Molecular permeability through polymer brush chains is implicated in surface lubrication, wettability, and solute capture and release. Probing molecular transport through polymer brushes can reveal information on the polymer nanostructure, with a permeability that is dependent on chain conformation and grafting density. Herein, we introduce a brush system to study the molecular transport of fluorophores from an aqueous droplet into the external “dry” polymer brush with the vapour phase above. The brushes consist of a random copolymer of *N*-isopropylacrylamide and a Förster resonance energy transfer (FRET) donor-labelled monomer, forming ultrathin brush architectures of about 35 nm in solvated height. Aqueous droplets containing a separate FRET acceptor are placed onto the surfaces, with FRET monitored spatially around the 3-phase contact line. FRET is used to monitor the transport from the droplet to the outside brush, and the changing internal distributions with time as the droplets prepare to recede. This reveals information on the dynamics and distances involved in the molecular transport of the FRET acceptor towards and away from the droplet contact line, which are strongly dependent on the relative humidity of the system. We anticipate our system to be extremely useful for studying lubrication dynamics and surface droplet wettability processes.

## 1. Introduction

Polymer brush surfaces offer intriguing functionality towards applications in switchable wettability [1], lubrication [2], controlled gating over nano- and micropores [3,4], and selective fouling [5]. A key feature that is important in this regard is the permeability of the brushes towards molecular transport [6]. The surfaces themselves consist of densely end-tethered polymer chains, with a permeability that is strongly dependent on chain conformation, between the extremes of stretched to fully collapsed. The combination of permeability and brush thickness has been referred to as the hydrodynamic fingerprint of the brush [7]. The ability of molecules to diffuse through a polymer brush is determined by multiple factors, including the conformation and density of the brush, the solvent content, the size of the molecules and the molecule–polymer interactions, amongst other contributions. Molecular transport through polymer brushes therefore offers an additional mechanism to study brush properties aside from only focusing on the ensemble average height of chains away from the anchoring surface. This allows a closer examination of the structure–property relationships of the brushes and ultimately a better understanding of their physical solvation.

Depending on the architecture of the system of interest, quantifying molecular transport through polymer brush layers can be relatively straightforward or tantalisingly difficult. Difficulty can come in terms of separating out contributions from transport above the brush (but in the hydration layer), at the interface, between the polymer chains, and at the anchoring surface, and for the case of “dry” brushes, from the vapour phase above the brush. Significant work has been conducted on understanding charge transport through zwitterionic brushes immobilized on conductive substrates [6,8,9], particularly by electrochemical impedance spectroscopy [10]. Elsewhere, silicon photonic microring resonators have been used to characterise analyte diffusion through a brush on a surface [11]. Recently, Masuda et al. [12] reported on self-oscillating polymer brushes by coupling the Belousov–Zhabotinsky (BZ) reaction to the changes in polymer brush conformation for binary brush systems, which highlighted the role of controlling transport through polymer brushes for the design of autonomous brush systems [13]. Alternatively, single-molecule fluorescence imaging and spectroscopic methods have provided fruitful details on molecular diffusion and interfacial dynamics in polymer brush thin films, particularly when the fluorescent species are bigger than solvent molecules and charges [14]. Of particular interest are fluorescence microscopy methods, which can be used to understand molecular transport in complex geometries (e.g., contacting surfaces) [15], with high sensitivity and, importantly, with spatially resolved resolutions. The ability of molecular fluorophores to penetrate polymer brushes has been studied by Pemberton and co-workers [16,17,18], who have reported on surface-tethered fluorophores for Förster resonance energy transfer (FRET), with polymer brushes grown above, via surface-initiated atom transfer radical polymerisation (SI-ATRP). These systems were subjected to laminar flow of aqueous solutions containing a second fluorophore for FRET; therefore, fluorescence microscopy imaging of the brush surfaces can be used to follow the laminar slip flow penetration (and thereby a view on the permeability) of the brushes. However, this system is limited to a view on transport to the directly anchoring surface (within the range of FRET), meaning that transport within the brush above the surface (i.e., away from the surface) is not accessible.

Inspired by the FRET approach, herein, we report a study on the molecular transport from aqueous droplets into new ultrathin polymer brush layers, which has been of recent interest for understanding surface wettability and brush solvation from direct liquid and vapours [19,20,21,22,23,24] and also in terms of more general polymer and small-molecule coatings [25,26]. We describe the assembly of FRET donor-containing ultrathin polymer brush surfaces composed of *N*-isopropylacrylamide (NIPAM), which were exposed to aqueous microdroplets containing FRET acceptors. The aim was to spatially resolve the molecular transport of the FRET acceptor into the brush surrounding the droplet by confocal laser scanning microscopy (CLSM). Ultrathin polymer brushes were chosen due to their uniformity in grafting structure and high grafting density, but also to minimise the distance over which molecular transport can occur through the polymer brush normal to the surface. This new approach allows for a direct probe of molecular transport through the brush, which has no dependence on surface–fluorophore contributions. Our results show that the FRET acceptor was transported through the polymer brush outside of the direct droplet, with a diffusion distance that is directly correlated to the relative humidity (RH) of the surrounding atmosphere, thereby highlighting how the external environment to the brush can control the molecular transport that occurs within it.

## 2. Results

### 2.1. Polymer Brush Assembly

**Figure 1:**
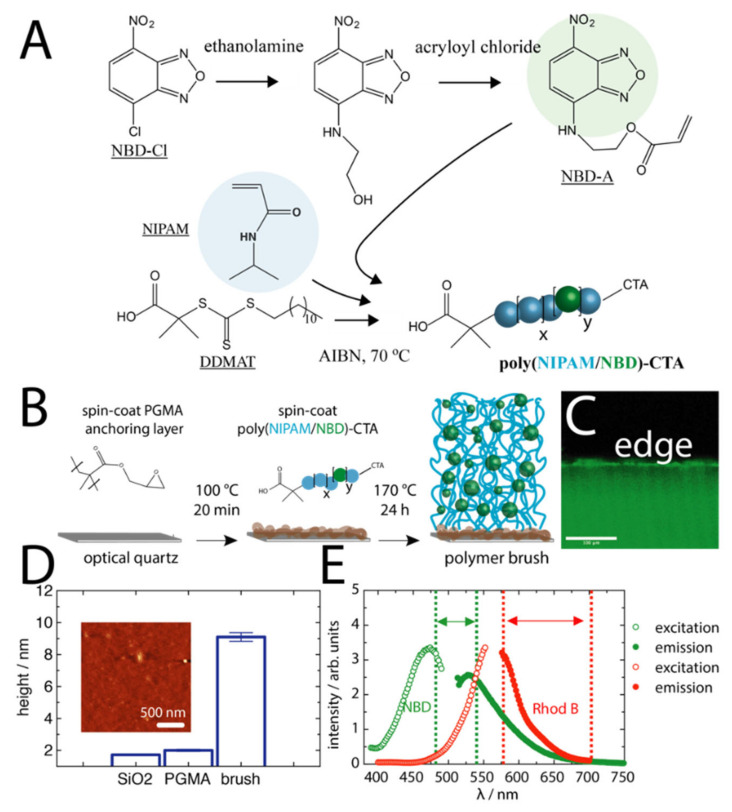
(**A**) Reaction schematic for the synthesis of the fluorophore monomer NBD-A (green sphere) along with its incorporation in random co-polymers with NIPAM (blue spheres) by RAFT polymerisation. (**B**) Reaction schematic for the assembly of poly(NIPAM/NBD) polymer brushes onto PGMA anchoring layers. (**C**) A confocal image insert of the edge of the polymer brush surface (excitation 458 nm, scale bar 100 μm). (**D**) Spectroscopic ellipsometry measurements of the heights of each layer (insert shows AFM topographic image). (**E**) Fluorescence excitation and emission spectra of the resulting NBD-labelled polymer brushes as well as that of Rhod B (in water), demonstrating spectral overlap. The dotted lines correspond to the spectral gating that was used for confocal analysis.

**Figure A1:**
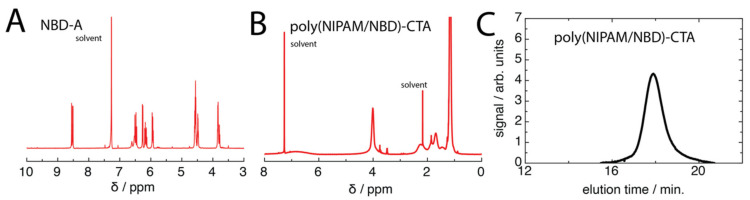
^1^H NMR spectra of the fluorescent monomer NBD-A (**A**) and the poly(NIPAM/NBD)-CTA (**B**) measured in CDCl_3_, and the GPC trace of poly(NIPAM/NBD)-CTA (**C**).

To assemble polymer brushes with an integrated FRET donor dye, we synthesised an acrylate derivative of 4-chloro-7-nitrobenzofurazan (NBD) for copolymerization with NIPAM. The NBD was modified through conjugation to ethanolamine followed by acryloyl chloride addition, yielding 4-[2-(acryloyloxy)ethylamino]-7-nitro-2,1,3-benzooxadiazole (NBD-A) (Figure 1A), which was confirmed by ^1^H NMR (Figure A1A). Upon this conjugation, the NBD motif became highly fluorescent, which is consistent with what is expected for secondary amine derivatives of NBD [27]. These typically have higher quantum yields in non-polar organic solvents than in water [28]. NBD-A was copolymerised with NIPAM via reversible addition–fragmentation chain transfer (RAFT) polymerisation from the chain transfer agent (CTA) 2-(dodecylthiocarbonothioylthio)-2-methylpropionic acid (DDMAT) (Figure A1B). This CTA was chosen for its carboxylic acid functionality to be used in subsequent polymer brush formation. The NIPAM monomer was used as the backbone for the polymer due to its exceptional responsiveness to temperature [29] and solvents [30] and for its ability to be assembled into dense polymer brush layers by a grafting-to approach [20,21,31]. The resulting polymers were bright yellow in appearance with a number average molecular weights of ~30 kDa (dispersity of 1.13) (Figure A1C), and UV-Visible spectroscopy analysis of the polymer against standard NBD solutions indicated an approximate ratio of 0.712 NBD molecules per single chain [21].

**Figure A2:**
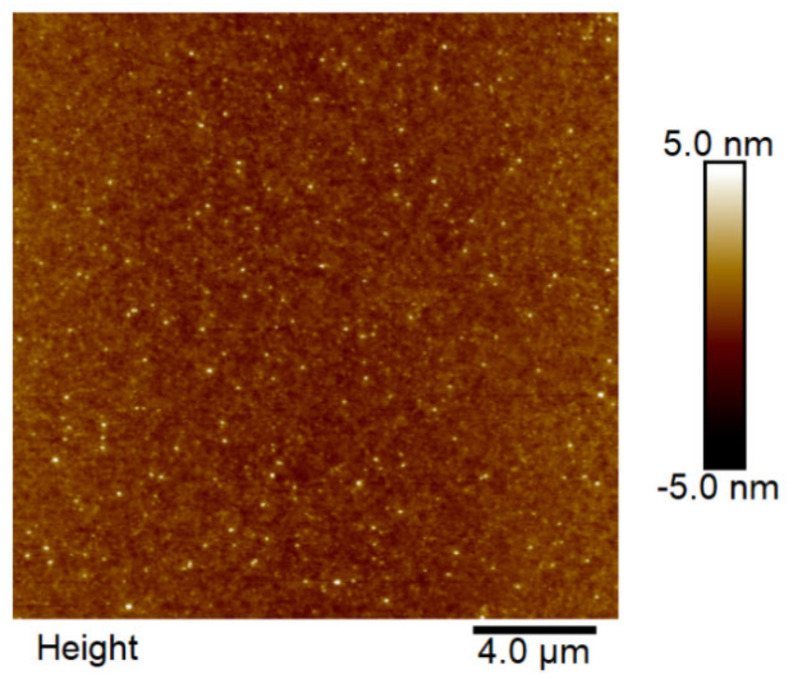
A larger AFM scan of the poly(NIPAM/NBD)-CTA polymer brush surface (complimentary to the insert in Figure 1D).

Using the carboxylic acid end group, the fluorescent polymers were assembled into polymer brush surfaces via a grafting-to approach onto macromolecular anchoring surfaces. This was achieved by first spin-coating a thin layer of poly(glycidyl methacrylate) (PGMA) onto surface-activated optical quartz surfaces (or silicon wafer substrates), followed by subsequent annealing in a vacuum oven for 20 min at 100 °C (Figure 1B). This process allows for a minor conjugation of the surface SiOH groups to the epoxide units of PGMA, forming ether linkages, with most epoxide groups remaining exposed. To the PGMA layer, the fluorescent polymer chains were spin-coated and allowed to conjugate for an extended period of time at 170 °C in a vacuum oven. The conjugation occured by an esterification of the DDMAT carboxylic acid end group of the polymer to the remaining active epoxide rings of the PGMA [21]. This process left the fluorescent polymer anchored to the PGMA layer, with the CTA group exposed to the environment above. The conjugation temperatures were chosen to be above the glass transition temperature of PNIPAM; therefore, the polymer chains were in a melt during the conjugation. Following subsequent washing, the surfaces exhibited clear fluorescence due to the integrated fluorophore (Figure 1C). The brush surfaces had a dry coating thickness of about 9 nm (measured by spectroscopic ellipsometry), with high surface homogeneity (Figure 1D, larger AFM scan in Figure A2). Importantly, the FRET donor dye (NBD) exhibited fluorescence emission properties that overlapped with the excitation properties of the FRET acceptor dye, rhodamine B (Rhod B) (Figure 1E). The grafting density of the polymer chains was determined from σ = (*H*_d_ ρ *N*_A_)/*M*_N_, where *H*_d_ is the dry brush thickness, ρ is the bulk density of the polymer (taken as 1.1 g cm^−3^) [20], and *N*_A_ is Avogadro’s number. The grafting density was found to be 0.20 chains/nm^2^, which is consistent with dense PNIPAM polymer brushes reported previously [20,21,31].

### 2.2. FRET of Aqueous Droplets on Polymer Brushes

**Figure 2:**
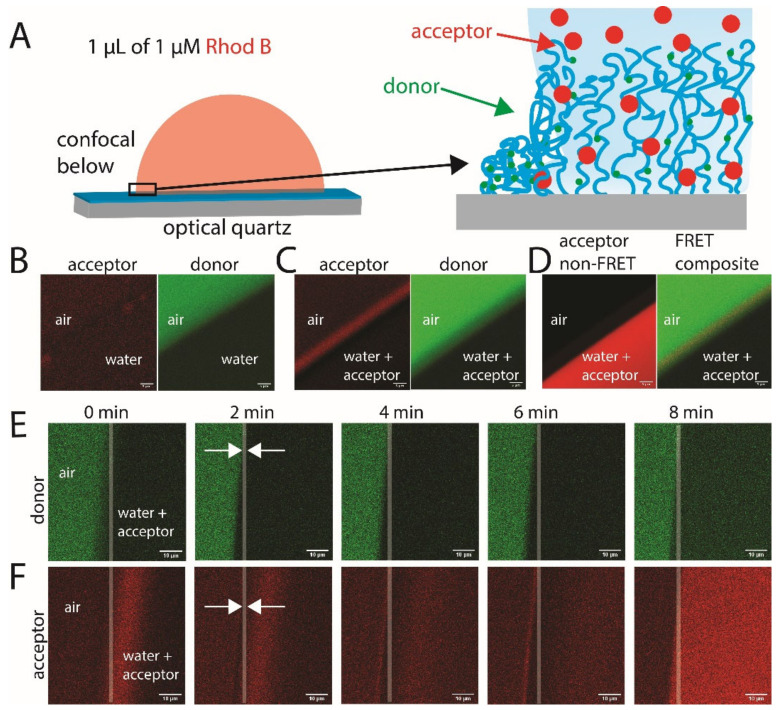
(**A**) Schematic of the experimental setup of a 1 μL aqueous droplet of 1 μM Rhod B (acceptor) on the poly(NΙPAM/NBD) polymer brush layers. (**B**) Confocal images (excitation: 458 nm, donor channel: 482–540 nm, acceptor channel: 578–703 nm) of the brush with a droplet of water. (**C**) An aqueous droplet containing 1 μM Rhod B after 5 min relaxation. (**D**) Direct excitation of the acceptor (non-FRET channel, excitation: 540 nm) and the composite of the FRET channels (excitation: 458 nm). The Rhod B droplet was then monitored as a function of time across the (**E**) donor and (**F**) acceptor channels. The arrows indicate the contact line, and the opaque white vertical lines are added to guide the eye between the donor and acceptor images. FRET excitation was performed at 458 nm at 21 °C and ~50% RH. Scale bars are 5 μm for (**B**–**D**) and 10 μm for (**E**,**F**).

The polymer brush surfaces had randomly distributed NBD fluorophores contained within a ~9 nm height when dry, and ~35 nm when solvated with good solvent (e.g., water). It is within this small 3D polymer space where we looked to understand the molecular transport of Rhod B through the polymer brushes at an aqueous droplet interface. Firstly, a test droplet of water was placed on the surface, where it was found that the donor channel was brightest in the dry area, but still fluorescent in the wet area underneath the droplet (Figure 2B). The difference in the NBD fluorescence between dry and wet is consistent with the reduction in the quantum yield of NBD in polar solvents [27]. However, for our polymer brush system, the difference in intensity between wet and dry was about ×1.8 times, whereas for free NBD, this difference is about ×20 times between water and non-polar organic solvents (e.g., toluene). This likely points towards an effect of the interaction between polymer and NBD, which reduces the interaction of NBD with water and its consequent impact on the quantum yield (the local concentration of NIPAM around the fluorophores is of the order of 2–3 M). Importantly, there was little fluorescence seen in the acceptor channel, although some was still present, indicating residual cross-talk between the channels.

**Figure A3:**
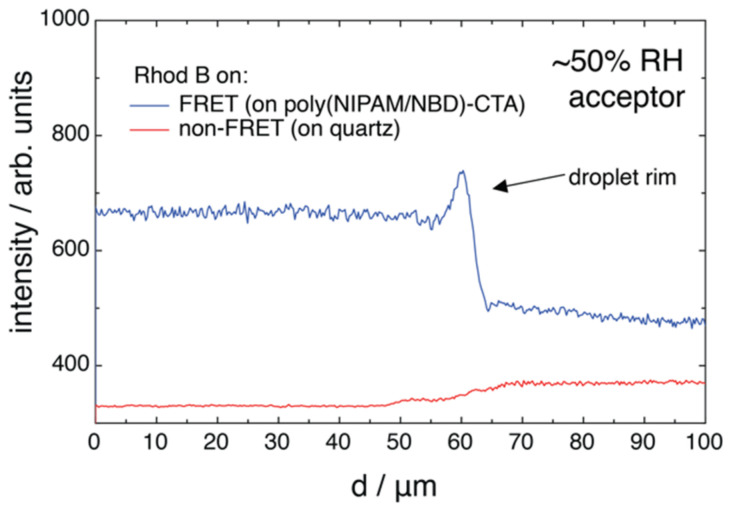
Comparison of line profiles across the acceptor channel of Figure 2C, compared to the same profile of a Rhod B drop placed on plain quartz (under same conditions).

A microdroplet of aqueous Rhod B solution (1 μM) was then placed onto a separate area of the brush (Figure 2A), with CLSM used to monitor the changes in donor and acceptor channel intensity upon donor excitation. After allowing the droplet to sit on the surface for 5 min, a clear increase in the acceptor channel was observed just outside of the droplet rim (Figure 2C), which was not seen for a droplet without a FRET acceptor (Figure 2B). The magnitude of the counts in this region was significantly larger than that of a Rhod B droplet on plain quartz (i.e., signal from scatter and direct Rhod B excitation) (Figure A3), demonstrating the FRET in this region. The signal measured at the acceptor channel results from FRET from the excited donor to the acceptor, occurring mainly at the droplet rim (Figure 2D). The Förster radius, *R*_0_, related to the range of FRET for the donor–acceptor pair, depends on the donor fluorescence yield, *φ*_f_, the refractive index of the medium, *n*, and the orientation factor, *κ*^2^. The orientation factor depends on the relative orientation of the transition dipole moments of the donor and acceptor, where an ensemble average of correlated donor–acceptor pairs gives $\langle \kappa^{2}\rangle$ = 2/3 if the molecules undergo fast rotational motion, or $\langle \kappa^{2}\rangle$ = 0.476 if the dipole moments are uncorrelated (no set orientations with respect to one another) or if they do not correlate on the time-scale of fluorescence. The *R*_0_ is given by [32].

(1) $$R_{0}{}^{6}=\frac{9000(\ln10)\kappa^{2}\varphi_{f}}{128\pi N_{A}n^{4}}\int_{0}^{\infty }F_{D}\left(\lambda \right)\varepsilon_{A}\left(\lambda \right)\lambda^{4}d\lambda$$

The above equation is in terms of the donor’s fluorescence spectrum, *F_D_*(λ), and the absorption spectrum of the acceptor $\varepsilon_{A}\left(\lambda \right)\;$(M^−1^ cm^−1^). Given that the *φ*_f_ of the NBD-integrated polymer brushes decreased by about ×1.8 in water, and that the refractive index increased by about ×1.3 (relative to air), the $R_{0}{}^{6}$ was expected to decrease by about ×6, which reduces the efficiency of energy transfer, given by *ET* =$\;R_{0}{}^{6}/\left(R_{0}{}^{6}+r^{6}\right)$, where *r* is the donor–acceptor distance between the donor and acceptor dipole centres. Therefore, the emission of the acceptor was mainly observed at the droplet interface region (the region the droplet solvates the brush outside the contact line), where the acceptor was at sufficiently low distances to the donors in the dry brush, as observed in Figure 2C. The Förster radius for the NBD-Rhod B pair was about *R*_0_ = 5 nm (in non-protic medium) [33], which means that FRET can only occur for donor–acceptor distances of up to a maximum of about 15 nm. Since the interface area showing the acceptor emission (~4 μm) was much larger than the experimental diffraction limit (CLSM resolution of about 300 nm), and given that the droplet was only (visibly) advancing before becoming pinned (i.e., not receding), this points towards the solvation of the brush outside of the aqueous droplet and thereby towards the molecular transport of Rhod B into the outside brush area.

To understand the transport process better, the FRET profiles were monitored as a function of time after the droplet was placed. The droplet was watched via a bright field until the droplet edge appeared to stabilise (i.e., to stop advancing), which occurred after approximately 5 s. Confocal images were then taken every 30 s until the droplet fully receded away (Figure 2E,F). We point out that each image was collected over the whole 30 s (i.e., *t* = 0 min means the image shows 0 min to 0.5 min). It was observed that, initially, at a distance about 5–10 μm behind the contact line (inside the droplet), there was a large increase in acceptor fluorescence, which then reduced in intensity with time and moved further towards the inside of the droplet. After about 2 min, the contact line became much more defined on both channels, likely indicating the point where the line had pinned the brush surface. Then, after about 4 min, it became clear that FRET was occurring just outside of the droplet in the vapour phase, where the droplet itself had not been previously. The corresponding acceptor emission grew stronger until the droplet began to recede away. Once the droplet had receded, the region that was wet before became drier, and the efficiency of FRET increased (increased brightness after 8 min in Figure 2E,F). This was to be expected, as the evaporation of droplets leaves a poorly hydrated, collapsed polymer brush with acceptors left by the receding fluid, with high FRET efficiency and corresponding suppression of donor emission. The dry brush region still showed higher emissions in the donor channel than the same area when wet, indicating a complex convolution of FRET from donor to acceptor and changing donor quantum yield depending on the solvation of the NBD fluorophores.

**Figure A4:**
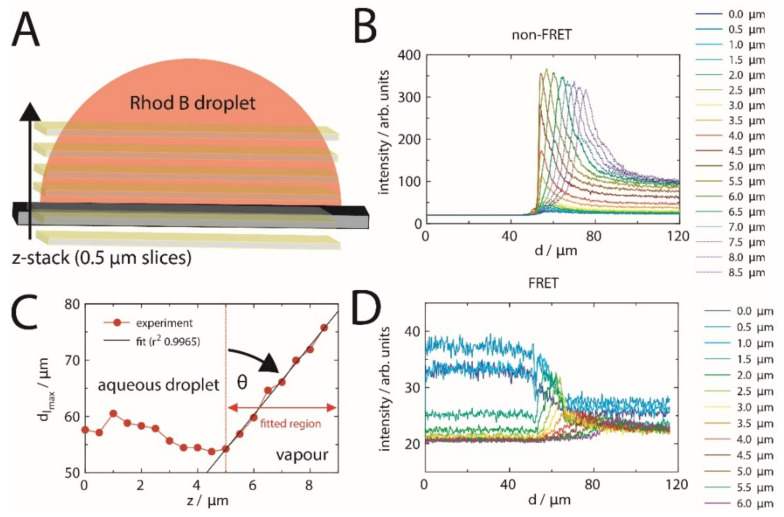
(**A**) A schematic describing the *z*-stacking of images up an aqueous droplet of Rhod B, with non-FRET (direct Rhod B excitation at 546 nm) line profiles (**B**), the subsequent analysis of the micro-contact angle from the shift in the position of the air–liquid interface peak in intensity (**C**), and (**D**) the line profile from FRET excitation (458 nm) for the same *z*-stack imaging. Note that the images were collected in rapid progression (~3 s per image), meaning that the intensity counts are lower than that measured for the 30 s images in the main text. Measurements were performed at 21 °C and ~50% RH.

We investigated the increased fluorescence on the inside of the droplet further by *z*-stacked imaging of the FRET channels (acceptor emission after donor excitation) and by non-FRET channels (direct acceptor excitation) transitioning up the droplet in the direction normal to the surface. The non-FRET channels revealed an increased concentration of Rhod B at the liquid–vapour interface going up the side of the droplet in the normal direction to the solid interface (Figure A1A). The profile was observed to shift towards the inside of the droplet as the *z* distance became greater, likely indicating an accumulation of Rhod B at the liquid–vapour interface. This confocal depth-scan of the side of the Rhod B droplet allowed the changing position of maximum intensity (in *x* or *y*) up the face of the droplet (in *z*) to be extracted, which was used to fit the contact angle of the droplet on the surface [34]. This was performed by linearly fitting the consistent change in the position of maximum intensity (i.e., past local surface effects) and by calculating the angle to the surface, which was found to yield about 44° (Figure A4C), which is consistent with that of other PNIPAM-copolymer surfaces under similar conditions [20]. Importantly, when the same z-stacking analysis was performed across the FRET channels, it was found that the intensity greatly reduced in moving away from the direct polymer brush surface (Figure A4D). However, a shift towards the inside of the droplet was still observed. This can be due to a couple of reasons: (1) possible bleed-through of donor emission to the acceptor channel, (2) lower wavelength excitation of the highly concentrated Rhod B at the interface, or (3) radiative energy transfer from NBD to Rhod B, which occurs over greater distances than FRET. Although it is difficult to say unequivocally at this point what the mechanism is, we anticipated channel bleed-through to be the key mechanism, still resulting from an increased concentration of Rhod B in the polymer brush layer behind the contact line in the droplet, which decreased significantly in magnitude upon transitioning up the droplet.

**Figure 3:**
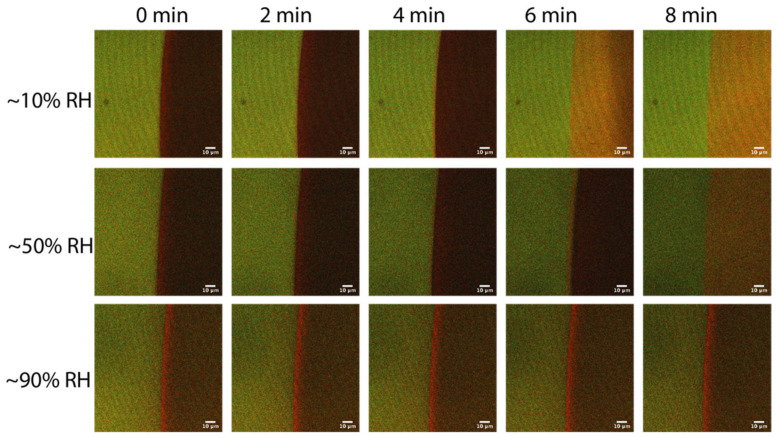
FRET-composite confocal images of a 1 μL aqueous droplet of 1 μM Rhod B (acceptor) on the polymer brush layers as a function of time (horizontal) and of relative humidity (RH) (vertical). FRET excitation was performed at 458 nm and at 21 °C. Scale bars are 10 μm.

**Figure A5:**
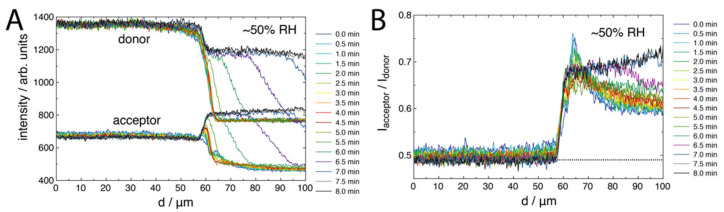
Acceptor and donor channel profiles (**A**), as well as the FRET-composites (**B**) of the confocal images in Figure 3 of the main text for the ~50% RH system. All parameters are the same as in Figure 4 of the main text.

To better understand the processes occurring at the droplet–brush interface, we performed a FRET analysis of the microdroplets as a function of relative humidity (RH), which allowed for control over the rate of droplet evaporation. This showed the effect of droplet drying and polymer brush hydration. The FRET composite images (Figure 3) allowed the changing distribution of acceptor signals to be spatially resolved around the interfacial region. For the ~10% RH system, the droplet could readily evaporate, fully receding by the 6 min stage. For the ~90% RH system, the droplet could not evaporate readily. Measuring at ~90% RH provided a view of the dynamics, as the droplet was somewhat pinned without receding. An interesting view of the spatial differences is, firstly, that the clear line of Rhod B outside of the droplet could only be seen for the ~10% RH and ~50% RH systems (Figure A5A), but not for the ~90% RH system. For this latter system, we instead observed a significantly stronger acceptor emission on and behind the contact line in the droplet, which did not appear to change in intensity or width over the entire time series studied.

**Figure 4:**
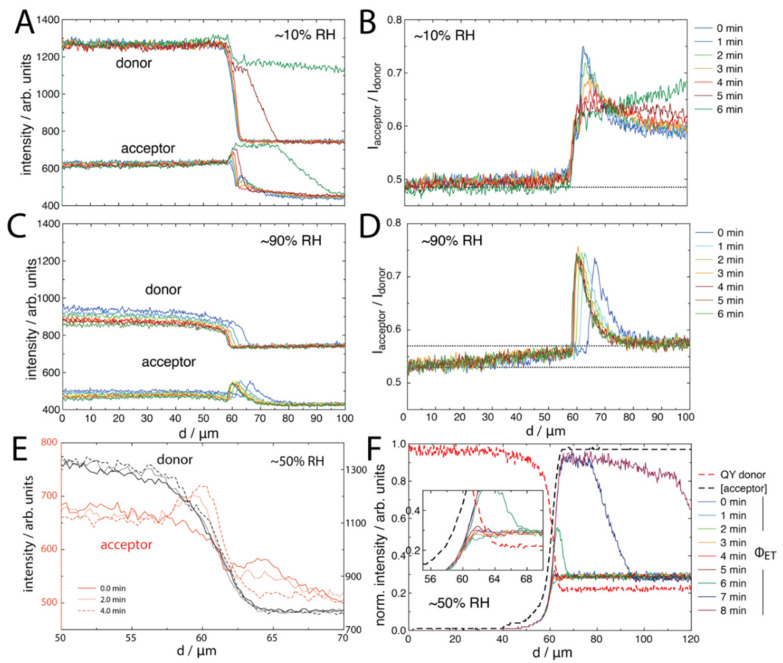
Acceptor and donor channel profiles, as well as FRET-composites of the confocal images in Figure 3: for (**A**,**B**), ~10% RH; for (**C**,**D**), ~90% RH, all given as a function of time (legend on right). The dotted lines indicate the approximate FRET baseline, which, for (**D**), is double, as the line has a clear gradient. (**E**) A zoom-in of the interfacial region of the ~50% RH system (acceptor left axis, donor right axis), and (**F**) calculated Φ_ET_ FRET profiles (normalized, given per time point), which are calculated from the quantum yield of the donor (QY donor, red dashed lines) and the concentration of acceptor ([acceptor], black dashed lines), shown for the ~50% RH system.

The data were analysed by extracting the line profiles of the separate donor and acceptor channels, as well as that of the acceptor:donor FRET ratio (Figure 4). What could be seen for the ~10% RH system was an increase in acceptor fluorescence behind the contact line in the droplet, as well as the emergence of a peak which appeared just on or outside of the contact line in the vapour phase. For the droplet with a contact angle of ~44°, most of the evaporation should occur at the contact line, creating a hydrodynamic flow towards the contact line. This likely leads to the enrichment of Rhod B towards the contact line. On the other hand, for the ~90% RH system, only an increase in acceptor emission was observed, just within the droplet, along with a slight advance in the contact line. Interestingly, we noted that the FRET profile for this system (Figure 4D) showed a gradient decreasing away from the droplet edge, suggesting that Rhod B had been transported outside of the droplet over large distances.

We focused on the increase in the acceptor emission at the droplet edge, as seen for the ~10% and ~50% RH systems. It was found that this increase in the acceptor emission overlaps with the decrease in fluorescence intensity of the donor as the brush transitions from a vapour to a liquid phase (Figure 4E) at the interface. This overlap was intriguing, as there were two competing components: (1) a decrease in the donor quantum yield from dry to wet environments, and (2) an increase in FRET efficiency as the Rhod B penetrated the brush in this region. We studied this mechanism by considering the balance of both these contributions at the interface.

The donor intensity as a function of time, *I_D_*(*t*), for FRET to a random distribution of acceptors can be given as a function of the donor fluorescence lifetime, *τ*_D_, as [32]

(2) $$I_{D}\left(t\right)=\exp\left(-\frac{t}{\tau_{D}}\right)\exp\left[-P{\left(\frac{t}{\tau_{D}}\right)}^{\beta }\right]$$

where *β* = *d*/6, *d* is the Euclidean dimension of the space in which the chromophores are distributed, and *P* is a parameter proportional to the local concentration of acceptors. The parameter *P* depends on the ensemble average correlation of dipole moments between donors and acceptors, *κ*^2^, as

(3) $$P=c_{\Delta }{\left(\frac{3\kappa^{2}}{2}\right)}^{\beta }\Gamma \left(1-\beta \right)$$

where $c_{\Delta }$ is the number of acceptors in a sphere of radius *R*_0_ and Γ is the Gamma function. We looked to determine the quantum yield of energy transfer, Φ_ΕΤ_, across the interfacial region, which can be written in terms of the fluorescence intensity of the donor in the presence, *F*_D_, and absence, *F*^0^_D_, of the FRET acceptor, as

(4) $$\Phi_{\mathrm{ET}}=1-\frac{F_{D}}{F^{0}{}_{D}}$$

This can be calculated from the integrals of the fluorescence decay with time, *t*, of the donor in the presence, *I*_D_, and absence, *I*^0^_D_, of the acceptor,

(5) $$\Phi_{\mathrm{ET}}=1-\frac{\int_{t}^{⬚}I_{D}\left(t\right)dt}{\int_{t}^{⬚}I^{0}{}_{D}\left(t\right)dt}$$

which integrates to [35]

(6) $$\Phi_{\mathrm{ET}}=\sqrt{\pi }\tau_{D}\left(\frac{P}{2}\right)\exp{\left(\frac{P}{2}\right)}^{2}\mathrm{erfc}\left(\frac{P}{2}\right)$$

If we allow $\langle \kappa^{2}\rangle$ = 2/3 (i.e., fast rotational motion) and consider that $c_{\Delta }\;$= 4/3 π *R*_0_^3^ [A] N_A_, Γ(0.5) = π^0.5^ and $R_{0}{}^{6}\propto \varphi_{D}/n^{4}$ (from Equation (1)), we obtain $P\propto \;\sqrt{\Phi_{D}/n^{4}}\left[A\right]$, where $\Phi_{D}$ is the donor quantum yield, and we can evaluate Equation (6) as

(7) $$\Phi_{\mathrm{ET}}=a_{1}a_{2}\sqrt{\Phi_{D}}\;\left[A\right]\times \exp{\left(a_{2}\sqrt{\Phi_{D}}\;\left[A\right]\right)}^{2}\mathrm{erfc}\left(a_{2}\sqrt{\Phi_{D}}\;\left[A\right]\right)$$

where *a*_1_ and *a*_2_ are constants. We can therefore calculate $\Phi_{\mathrm{ET}}$ from knowledge of only $\Phi_{D}$ and the concentration of the acceptor, [A], as a function of distance normal to the interface, *d*. For the $\Phi_{D}$, we used the donor emission with the dark current subtracted (signal from scatter into a plain quartz surface at the same focal length). For [A], we inverted the donor emission profile, which assumes that the droplet interface follows the droplet shape across the donor profile and that the acceptor is transported in this same solvation shape. Both quantities were normalised for direct comparison. Using this simple two variable model, we found that the peak in the FRET at the interface was reproduced for the ~50% RH system (Figure 4F) as well as for the increased FRET efficiency as the droplet receded with time. The ~50% RH was used for the model input due to the slower change in Rhod B distributions (compared to the ~10% RH system, as example). This shows that the FRET signal observed at the contact line was due to a balance between donor quantum yield decreasing and due to increasing the acceptor emission as more acceptor molecules were transported to the contact line region.

**Figure 5:**
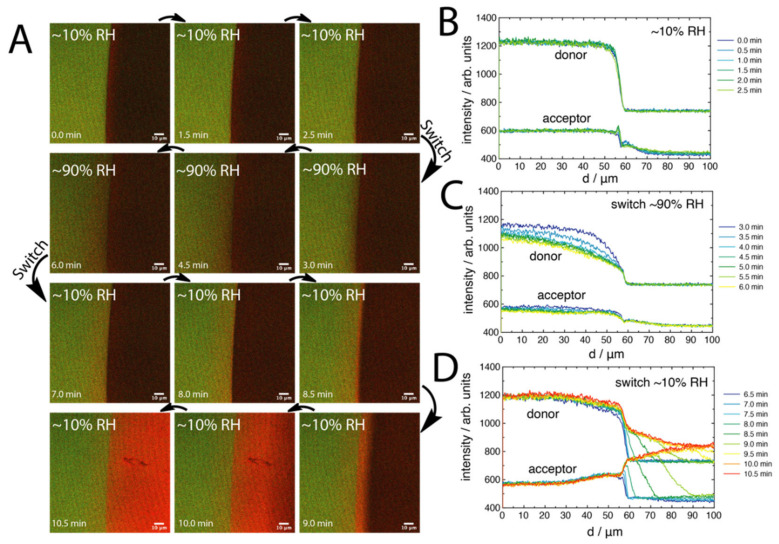
FRET-composite confocal images of a 1 μL aqueous droplet of 1 μM Rhod B (acceptor) on the polymer brush layers as a function of time (**A**). The RH was switched after 2.5 min from ~10% RH to ~90% RH and switched back to ~10% RH after 6.5 min. Line profiles for the donor and acceptor channels are shown for each of the switches in RH (**B**–**D**). Note that all measurements are continuous, with only the RH changed. FRET excitation was performed at 458 nm and at 21 °C. The arrows indicate the direction of the time series. Scale bars are 10 μm.

For the ~90% RH system, it was noted that the donor emission (Figure 4C) was significantly decreased in comparison to the other systems. Specifically, the emission was about 900 counts, compared to about 1300 counts. We hypothesised that this was due to both the solvation of the polymer brush outside of the droplet at high humidity, as well as some possible FRET from Rhod B transported outside of the droplet region. Previously, solvation has been observed from outside of aqueous droplets on similar brush surfaces [20]. To clarify this, we designed “switch” experiments, whereby an acceptor droplet was placed on the polymer brush at ~10% RH. Then, after 2.5 min, the system was switched to ~90% RH (Figure 5A). The switch was performed before the droplet had the opportunity to recede at ~10% RH. It was seen that the initial contact line peak in the acceptor emission emerged by 2.5 min at ~10% RH. This quickly vanished upon switching to ~90% RH. It appeared that, after the switch, some of the acceptors had diffused further past the contact line into the brush in contact with the vapour phase, with a simultaneous drop in donor intensity across this same region. Then, upon recycling to ~10% RH after 6.5 min, the polymer brush in contact with the vapour phase quickly regained intensity while also showing a more distinctive acceptor emission, before the droplet fully receded, leaving behind a stronger acceptor emission due to higher FRET efficiency. The line profile analysis of the first ~10% RH stage (Figure 5B) showed similar features to Figure 4A. However, upon switching to ~90% RH, the line profiles (Figure 5C) showed an interesting decrease in donor intensity as a function of time, which was a smooth transition towards the vapour phase at larger distances (transition spanning over 60 μm from the contact line). The acceptor profile, on the other hand, showed that the acceptor peak at the contact line had disappeared, but other features seemed constant. This potentially indicates a further convolution of the reducing quantum yield of NBD (the brush may be wet outside of the droplet due to the high humidity) along with Rhod B transported outside of the droplet. This was clarified by analysing the profiles after the switch back to ~10% RH (Figure 5D), where the donor profile increased in intensity back towards the starting level, but the acceptor profile suddenly showed a distinctive “hump” that spanned about 30 μm away from the droplet edge. This was the region over which Rhod B was transported through the polymer brush by the droplet solvation front upon switching the humidity.

**Figure 6:**
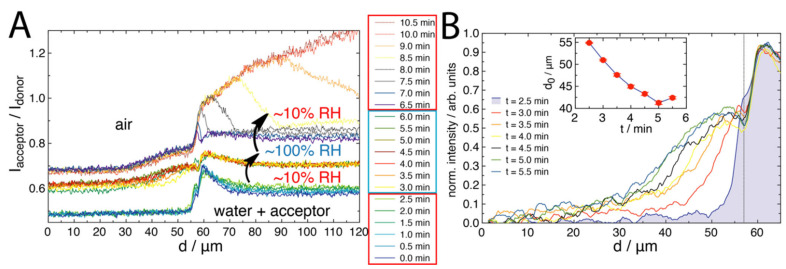
(**A**) FRET-composite profiles of confocal images in Figure 5, where the ~90% RH and final ~10% RH profiles are offset vertically for clarity by 0.1 and 0.2, respectively. The dotted lines indicate profiles that are across the receding droplet (i.e., where the surface was drying). (**B**) A zoom-in of the ~90% RH FRET profile that is normalized at d = 0 and by intensity for comparison purposes. The insert shows an estimate of the position of the dividing interface, d_0_, of each of the profiles, obtained from fitting a hyperbolic tangential decay [37], performed to highlight the movement of the FRET profile (Figure A6). The last ~10% RH profile (2.5 min) is coloured blue for distinction.

**Figure A6:**
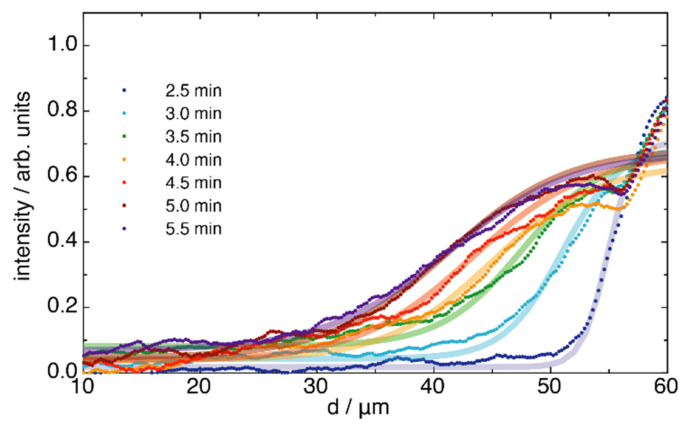
A comparison of the fits (solid lines) to the data in Figure 6B (dots) in order to extract the approximate position of the dividing interface, *d*_0_, of the decay in intensity, *I*, outside of the droplet. The fits have a hyperbolic tangent form of $I=\frac{1}{2}\left(\rho_{L}+\rho_{V}\right)-\frac{1}{2}\left(\rho_{L}-\rho_{V}\right)\tanh\left[\frac{d-d_{0}}{h}\right]$, where the fitted parameter *ρ_ι_* corresponds to the density of counts in the *i*th phase and where *h* corresponds to the thickness of the interface region [37]. Note that the fits cannot capture the whole intensity fluctuation across the interface, but they capture that of the secondary decay outside of the droplet edge.

**Figure A7:**
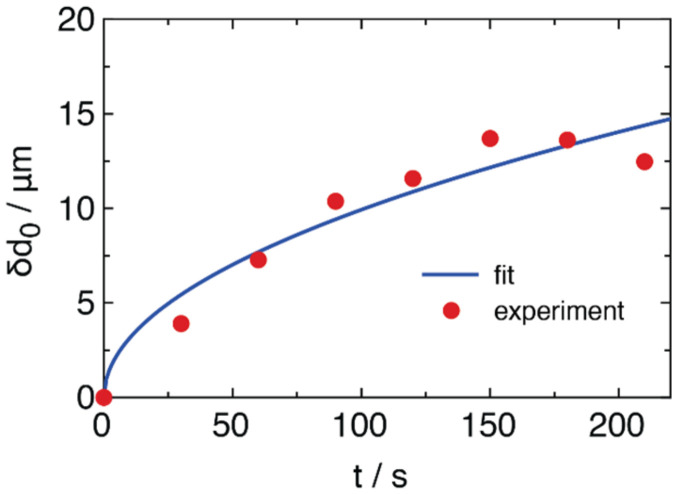
A re-arrangement of the change in the fitted dividing interface (Figure A6) against time in order to fit an approximate function to describe a quasi-diffusion rate of Rhod B, given as $\delta d_{0}=\sqrt{2\;D\;t}$, where *D* is the fitted diffusion coefficient, which yields 4.9 cm^2^/s. We point out that this neglects the changing concentrations of water and diffusion rates as a function of distance from the contact line, and they should only serve as very basic indicators of the diffusion rate of Rhod B.

The transport of Rhod B outside of the droplet became clearer when analysing the FRET profile for the ~90% RH system after the initial switch (Figure 6A), where the “hump” was clearly seen. This was compared further in Figure 6B by normalising the profiles with the last ~10% RH before the switch occurred (Figure A6). What was found was a Fickian-like diffusion profile of the acceptor emission due to FRET extending away from the contact line with time. After 2.5 min at ~90% RH, the solvation front appeared to stabilize at a distance of about 35 μm from the initial front. This is in excellent agreement with other work on mechanofluorescent surfaces that were used to study droplet swelling dynamics on polymer brushes [20]. We could fit the changing profiles of Figure 6B to obtain a quasi-diffusion coefficient for the Rhod B front, *D*, which yielded 4.9 cm^2^/s (Figure A7), which is slower than RhodB in water [36] but is consistent with other thin polymer films [15] and significantly faster than much thicker polymer brushes [18]. Note that our value is approximate, which neglects other contributing factors. Interestingly, upon the final switch back to ~10% RH and allowing the droplet to recede, we observed that FRET was significantly more efficient than that observed for the single-condition droplet swelling study (compare Figure 4B and Figure 6A). This intriguing result suggests that, by switching the RH, which causes the transport of Rhod to rapidly change, Rhod B had penetrated further into the polymer brush after the droplet had evaporated and fully receded. This switch-dependent transport of the Rhod B through the brush is the subject of a future study.

Our results show that the molecular transport of fluorophores can be monitored around the three-phase contact line of an aqueous droplet on a polymer brush. This reveals information on the humidity-controlled diffusion into the polymer brush, in terms of both dynamics and distances. We anticipate an exciting extension of this method to be monitoring the molecular transport through patterned [38] binary brush systems under different stimuli that affect each pattern’s chain conformation.

## 3. Materials and Methods

### 3.1. Materials

All chemicals were of analytical grade and used as received without purification, with the exception of *N*-isopropylacrylamide (NIPAM), which was purified by recrystallisation from hexane (×2). High-purity water (Milli-Q water) with a resistivity of >18.2 MΩ cm was obtained from an inline Millipore RiOs/Origin water purification system (Millipore Corporation, St. Louis, MA, USA). Polished single-crystal (100)-silicon wafers were obtained from Silicon Materials, Kaufering, Germany, with a native SiO_2_ layer thickness of ~1.4 nm. Optical fused quartz square cover slips (22 × 22 × 0.2 mm) were obtained from Micro to Nano (Haarlem, The Netherlands). PGMA (*M*_N_ = 15,000 g mol^−1^, Ð = 1.6) was obtained from Polymer Source Inc. (Montreal, QC, Canada). 4-Chloro-7-nitrobenzofurazan (NBD-Cl), ethanolamine, acetonitrile (ACN), acryloyl chloride, thionyl chloride, dichloromethane (DCM), NIPAM, azobisisobutynitrile (AIBN), 1,4-dioxane (anhydrous), and diethyl ether were obtained from Sigma-Aldrich. THF was obtained from Acros Organics. Chloroform was obtained from Fisher Chemicals. CDCl_3_ and DMSO-*d*_6_ were obtained from Eurisotop (Saint-Aubin, France).

### 3.2. Synthesis of NBD-A

NBD-A was synthesised as reported previously [21]. The ^1^H NMR spectra are shown in Figure A1A.

### 3.3. RAFT Polymer Synthesis

The NIPAM/NBD copolymer was synthesised as reported previously [21]. The ^1^H NMR spectra are shown in Figure A1B, and the GPC trace is shown in Figure A1C.

### 3.4. Grafting-To of Polymer Brushes

Quartz cover slips (22 × 22 × 0.2 mm) and silicon wafers (20 × 15 mm) were sonicated in ethanol for 20 min at 37 °C and then dried under a stream of nitrogen. The substrates were then subjected to oxygen plasma for 90 s (Harrick, Plasma Cleaner PDC-002 with Plasma Flo PFC-FMG). In order to obtain an anchoring layer for the subsequent grafting-to process of the RAFT copolymer, a filtered solution of PGMA (80 μL (silicon wafer) or 160 μL (quartz) of a 0.2 mg mL^−1^ solution in chloroform) was spin-coated (Spin150 spin coater, Polos, *v* = 2000 r min^−1^, *a* = 1000 r (min s)^−1^, *t* = 10 s) onto the activated substrates. The PGMA anchoring layer was subsequently annealed at 100 °C in a vacuum oven for 20 min to react the silanol groups of the substrate with a fraction of the epoxy group of the PGMA. The remaining epoxy groups were then used for the subsequent grafting-to process. A filtered solution of RAFT co-polymer (120 μL (silicon wafer) or 240 μL (quartz) of a 11 mg mL^−1^ solution) was subsequently spin-coated on the substrates (Spin150 spin coater, Polos, *v* = 2000 r min^−1^, *a* = 1000 r (min s)^−1^, *t* = 10 s), followed by annealing at 170 °C in a vacuum oven for 24 h. To remove non-covalently bound polymers, the resulting substrates were firstly immersed in Milli-Q water, then extracted in Milli-Q water overnight, and then rinsed with EtOH and dried under a stream of nitrogen.

### 3.5. Fluorescence Measurements

Fluorescence measurements were performed with a multimode microplate reader (Tecan Spark 10M, Männedorf, Switzerland). Measurements were performed in Milli-Q water at 23 °C.

### 3.6. Atomic Force Microscopy (AFM)

In-air tapping mode AFM was performed with a Nanoscope Dimension D3100 V (Veeco Instruments, Plainview, NY, USA) with software analysis with NanoScope Analysis 1.7 and a phase tip (500 kHz, *k* = 3–4 N m^−1^).

### 3.7. Spectroscopic Ellipsometry

The ellipsometry measurements were conducted at a constant temperature of 21 °C in filtered Milli-Q water. The measurements and analyses were conducted as reported previously [20].

### 3.8. Confocal Laser Scanning Microscopy (CLSM)

A combination setup of an Axio Observer Z.1 inverted microscope with an LSM710 confocal laser scanning module (Carl Zeiss Microscopy, Oberkochen, Germany) was used. Measurements were performed with either a 10× or 50× in-air objective. The donor was excited with an argon laser (458 nm) and the acceptor (for non-FRET measurements) with a helium–neon laser (543 nm). Unless otherwise stated, the pin-hole was set at 1.4 μm, with a laser intensity of 3% and a gain of 750. The donor collection window was 482–540 nm, and the acceptor was 578–703 nm. A dark (no-collection) window was kept at 38 nm in order to avoid crosstalk between channels as much as possible. Unless otherwise stated, the conditions were maintained at 21 °C and ~50% RH. Before measurements, the polymer brush surfaces were quickly cleaned with ethanol and then dried under a stream of nitrogen, and they were placed on the sample holder. The ideal *z*-position was then found with respect to the maximum fluorescence of the FRET donor (NBD). For droplet studies, the setup started with bright field observation while the droplet was placed. This allowed the droplet edge to be rapidly found and positioned in the field-of-view. Once this was positioned, the contact line was monitored briefly for the initial advancing movement. Once this stablised (~5 s), the system was initiated in CLSM mode. For time-separated measurements, time series were performed sequentially at 30 s intervals. Images were collected over the entire 30 s between measurements. In-text, the time refers to the initial point in the measurement (i.e., time 0.5 min is for measurements started at 0.5 min, which proceeded over the subsequent 30 s). For humidity control (~10% and ~90% RH), a stream of nitrogen gas was split and controlled by two flow controllers from ANALYT-MTC Meßtechnik GmbH (Müllheim, Germany). One stream was saturated with water by guiding it through gas wash bottles (DURAN Group GmbH, Wertheim, Germany). When the flow rates of the two streams were varied, the RH of the united stream could be controlled.

## 4. Conclusions

Understanding molecular transport through polymer brushes is needed to leverage their use as selectively permeable gatings, switchable lubrication, and smart, functional surface coatings. Herein, we devised a system of PNIPAM-based polymer brush layers, which contained covalently tethered FRET donor molecules at random locations on the chains. This surface was used to study the molecular transport of Rhod B (FRET acceptor) from an aqueous droplet into the polymer brush, at the three-phase contact line. We found primarily an accumulation of Rhod B at the contact line, which could be observed by CLSM. The mechanism of FRET at and outside of the contact line was understood to be a convolution of the reducing quantum yield of the donor in transitioning from a dry to a wet environment, with an increase in the concentration of acceptors in the contact line region. This accumulation of acceptor dye was strongly dependent on the relative humidity (RH), where, at ~10% RH, the Rhod B was primarily limited to within 5 μm of the contact line region in the vapour phase. However, at ~90% RH, the Rhod B was rapidly transported outwards, away from the droplet, and through the polymer brush, reaching distances upwards of 35 μm away from the contact line. This transport followed a typical diffusion-like behaviour, ultimately highlighting the length scales involved in the solvation of the polymer brush in regions outside of the aqueous droplet (with solvent from within the droplet). We anticipate our method to have application in better understanding the lubricity and wettability of polymer brush surfaces, solute transport and concentration at the brush, and ultimately leading towards devising new fluorescence-based communicative polymer brush systems.

## Data Availability

The data presented in this study are available on request from the corresponding author.

## References

[1] Kobayashi M., Terayama Y., Yamaguchi H., Terada M., Murakami D., Ishihara K., Takahara A. (2012). Wettability and antifouling behavior on the surfaces of superhydrophilic polymer brushes. Langmuir.

[2] Kobayashi M., Takahara A. (2010). Tribological properties of hydrophilic polymer brushes under wet conditions. Chem. Rec..

[3] De Groot G.W., Santonicola M.G., Sugihara K., Zambelli T., Reimhult E., Voros J., Vancso G.J. (2013). Switching transport through nanopores with pH-responsive polymer brushes for controlled ion permeability. ACS Appl. Mater. Interfaces.

[4] Speyer K., Pastorino C. (2019). Pressure responsive gating in nanochannels coated by semiflexible polymer brushes. Soft Matter.

[5] Ng G., Li M., Yeow J., Jung K., Pester C.W., Boyer C. (2020). Benchtop preparation of polymer brushes by SI-PET-RAFT: The effect of the polymer composition and structure on inhibition of a Pseudomonas biofilm. ACS Appl. Mater. Interfaces.

[6] Motornov M., Sheparovych R., Katz E., Minko S. (2008). Chemical gating with nanostructured responsive polymer brushes: Mixed brush versus homopolymer brush. ACS Nano.

[7] Youssef M., Morin A., Aubret A., Sacanna S., Palacci J. (2020). Rapid characterization of neutral polymer brush with a conventional zetameter and a variable pinch of salt. Soft Matter.

[8] Whiting G.L., Snaith H.J., Khodabakhsh S., Andreasen J.W., Breiby D.W., Nielsen M.M., Greenham N.C., Friend R.H., Huck W.T.S. (2006). Enhancement of charge-transport characteristics in polymer films using polymer brushes. Nano Lett..

[9] Yameen B., Ali M., Neumann R., Ensinger W., Knoll W., Azzaroni O. (2009). Single Conical Nanopores Displaying pH-Tunable Rectifying Characteristics. Manipulating Ionic Transport with Zwitterionic Polymer Brushes. J. Am. Chem. Soc..

[10] Anthi J., Kolivoska V., Holubova B., Vaisocherova-Lisalova H. (2021). Probing polymer brushes with electrochemical impedance spectroscopy: A mini review. Biomater. Sci..

[11] Wetzler S.P., Miller K.A., Kisley L., Stanton A.L.D., Braun P.V., Bailey R.C. (2020). Real-time measurement of polymer brush dynamics using silicon photonic microring resonators: Analyte partitioning and interior brush kinetics. Langmuir.

[12] Masuda T., Hidaka M., Murase Y., Akimoto A.M., Nagase K., Okano T., Yoshida R. (2013). Self-oscillating polymer brushes. Angew. Chem. Int. Ed. Engl..

[13] Conrad J.C., Robertson M.L. (2019). Towards mimicking biological function with responsive surface-grafted polymer brushes. Curr. Opin. Solid State Mater. Sci..

[14] Wang S., Jing B., Zhu Y. (2014). Molecule motion at polymer brush interfaces from single-molecule experimental perspectives. J. Polym. Sci. B Polym. Phys..

[15] Frost R., Debarre D., Jana S., Bano F., Schunemann J., Gorlich D., Richter R.P. (2020). A method to quantify molecular diffusion within thin solvated polymer films: A case study on films of natively unfolded Nucleoporins. ACS Nano.

[16] Wang H., Cheng L., Saez A.E., Pemberton J.E. (2015). Flow Field penetration in thin nanoporous polymer films under laminar flow by Förster resonance energy transfer coupled with total internal reflectance fluorescence microscopy. Anal. Chem..

[17] Wang H., Pemberton J.E. (2017). Effect of solvent quality on laminar slip flow penetration of poly(*N*-isopropylacrylamide) films with an exploration of the mass transport mechanism. Langmuir.

[18] Wang H., Pemberton J.E. (2019). Direct nanoscopic measurement of laminar slip flow penetration of deformable polymer brush surfaces: Synergistic effect of grafting density and solvent quality. Langmuir.

[19] Schubotz S., Honnigfort C., Nazari S., Fery A., Sommer J.U., Uhlmann P., Braunschweig B., Auernhammer G.K. (2021). Memory effects in polymer brushes showing co-nonsolvency effects. Adv. Colloid Interface Sci..

[20] Besford Q.A., Merlitz H., Schubotz S., Yong H., Chae S., Schnepf M.J., Weiss A.C.G., Auernhammer G.K., Sommer J.U., Uhlmann P. (2022). Mechanofluorescent polymer brush surfaces that spatially resolve surface solvation. ACS Nano.

[21] Besford Q.A., Yong H., Merlitz H., Christofferson A.J., Sommer J.U., Uhlmann P., Fery A. (2021). FRET-integrated polymer brushes for spatially resolved sensing of changes in polymer conformation. Angew. Chem. Int. Ed. Engl..

[22] Shiomoto S., Higuchi H., Yamaguchi K., Takaba H., Kobayashi M. (2021). Spreading dynamics of a precursor film of ionic liquid or water on a micropatterned polyelectrolyte brush surface. Langmuir.

[23] Leong F.Y., Le D.-V. (2021). Dynamics of a droplet on a polymer brush in channel flow. Phys. Fluids.

[24] Ritsema van Eck G.C., Chiappisi L., de Beer S. (2022). Fundamentals and applications of polymer brushes in air. ACS Appl. Polym. Mater..

[25] Eggenberger O.M., Ying C., Mayer M. (2019). Surface coatings for solid-state nanopores. Nanoscale.

[26] Khalil A., Rostami P., Auernhammer G.K., Andrieu-Brunsen A. (2021). Mesoporous coatings with simultaneous light-triggered transition of water imbibition and droplet coalescence. Adv. Mater. Interfaces.

[27] Fery-Forgues S., Fayet J.P., Lopez A. (1993). Drastic changes in the fluorescence properties of NBD probes with the polarity of the medium: Involvement of a TICT state?. J. Photochem. Photobiol. A Chem..

[28] Qiao J., Chen C., Qi L., Liu M., Dong P., Jiang Q., Yang X., Mu X., Mao L. (2014). Intracellular temperature sensing by a ratiometric fluorescent polymer thermometer. J. Mater. Chem. B.

[29] Yu Y., Kieviet B.D., Liu F., Siretanu I., Kutnyanszky E., Vancso G.J., de Beer S. (2015). Stretching of collapsed polymers causes an enhanced dissipative response of PNIPAM brushes near their LCST. Soft Matter.

[30] Yong H., Bittrich E., Uhlmann P., Fery A., Sommer J.-U. (2019). Co-nonsolvency transition of poly(*N*-isopropylacrylamide) brushes in a series of binary mixtures. Macromolecules.

[31] Rauch S., Eichhorn K.-J., Oertel U., Stamm M., Kuckling D., Uhlmann P. (2012). Temperature responsive polymer brushes with clicked rhodamine B: Synthesis, characterization and swelling dynamics studied by spectroscopic ellipsometry. Soft Matter.

[32] Farinha J.P.S., Martinho J.M.G. (2008). Resonance energy transfer in polymer nanodomains. J. Phys. Chem. C.

[33] Pathak P., London E. (2011). Measurement of lipid nanodomain (raft) formation and size in sphingomyelin/POPC/cholesterol vesicles shows TX-100 and transmembrane helices increase domain size by coalescing preexisting nanodomains but do not induce domain formation. Biophys. J..

[34] Farinha J.P.S., Winnik M.A. (1999). Imaging an oil droplet under a latex film. Langmuir.

[35] Farinha J.P.S., Vorobyova O., Winnik M.A. (2000). An energy tranfer study of the interface thickness in blends of poly(butyl methacrylate) and poly(2-ethylhexyl methacrylate). Macromolecules.

[36] Gendron P.O., Avaltroni F., Wilkinson K.J. (2008). Diffusion coefficients of several rhodamine derivatives as determined by pulsed field gradient-nuclear magnetic resonance and fluorescence correlation spectroscopy. J. Fluoresc..

[37] Besford Q.A., Liu M., Christofferson A.J. (2018). Stabilizing dipolar interactions drive specific molecular structure at the water liquid-vapor interface. J. Phys. Chem. B.

[38] Poisson J., Polgar A.M., Fromel M., Pester C.W., Hudson Z.M. (2021). Preparation of patterned and multilayer thin films for organic electronics via oxygen-tolerant SI-PET-RAFT. Angew. Chem. Int. Ed. Engl..

